# Distribution Characteristics of Soil Viruses Under Different Precipitation Gradients on the Qinghai-Tibet Plateau

**DOI:** 10.3389/fmicb.2022.848305

**Published:** 2022-04-07

**Authors:** Miao-Miao Cao, Si-Yi Liu, Li Bi, Shu-Jun Chen, Hua-Yong Wu, Yuan Ge, Bing Han, Li-Mei Zhang, Ji-Zheng He, Li-Li Han

**Affiliations:** ^1^State Key Laboratory of Urban and Regional Ecology, Research Center for Eco-Environmental Sciences, Chinese Academy of Sciences, Beijing, China; ^2^University of Chinese Academy of Sciences, Beijing, China; ^3^The Zhongke-Ji’an Institute for Eco-Environmental Sciences, Ji’an, China; ^4^Faculty of Veterinary and Agricultural Sciences, The University of Melbourne, Parkville, VIC, Australia; ^5^Information Technology Center, Tsinghua University, Beijing, China; ^6^State Key Laboratory of Soil and Sustainable Agriculture, Institute of Soil Science, Chinese Academy of Sciences, Nanjing, China; ^7^Key Laboratory for Humid Subtropical Eco-Geographical Processes of the Ministry of Education, Fujian Normal University, Fuzhou, China

**Keywords:** soil viruses, metagenome, precipitation, abundance, diversity, carbon cycle

## Abstract

Viruses are extremely abundant in the soil environment and have potential roles in impacting on microbial population, evolution, and nutrient biogeochemical cycles. However, how environment and climate changes affect soil viruses is still poorly understood. Here, a metagenomic approach was used to investigate the distribution, diversity, and potential biogeochemical impacts of DNA viruses in 12 grassland soils under three precipitation gradients on the Qinghai-Tibet Plateau, which is one of the most sensitive areas to climate change. A total of 557 viral operational taxonomic units were obtained, spanning 152 viral families from the 30 metagenomes. Both virus-like particles (VLPs) and microbial abundance increased with average annual precipitation. A significant positive correlation of VLP counts was observed with soil water content, total carbon, total nitrogen, soil organic matter, and total phosphorus. Among these biological and abiotic factors, SWC mainly contributed to the variability in VLP abundance. The order *Caudovirales* (70.1% of the identified viral order) was the predominant viral type in soils from the Qinghai-Tibet Plateau, with the *Siphoviridae* family being the most abundant. Remarkably, abundant auxiliary carbohydrate-active enzyme (CAZyme) genes represented by glycoside hydrolases were identified, indicating that soil viruses may play a potential role in the carbon cycle on the Qinghai-Tibet Plateau. There were more diverse hosts and abundant CAZyme genes in soil with moderate precipitation. Our study provides a strong evidence that changes in precipitation impact not only viral abundance and virus–host interactions in soil but also the viral functional potential, especially carbon cycling.

## Introduction

Viruses are the most abundant lifeform on Earth and highly encompass their biodiversity (Paez-Espino et al., 2016). Previous marine viral ecology studies demonstrated that viruses play a crucial part in the environment. Firstly, viruses can have an influence on microbial populations and evolution by modulating and controlling the abundance, diversity, and functional processes of a host through cell lysis (Thingstad, 2000) or lysogeny (Knowles et al., 2016). Two models were built to explain the viruses’ role on microbial populations. For example, the “kill-the-winner” (KtW) model of lytic infection predicts that density- and frequency-dependent viral predation suppresses the blooms of rapidly growing hosts, maintaining and increasing the stability and diversity of host communities (Thingstad and Lignell, 1997). In contrast, the “piggyback-the-winner” (PtW) model of lysogenic infection predicts that increasing host density will enhance lysogenic incidence (i.e., “more microbes, fewer viruses”) (Knowles et al., 2016).

Secondly, viruses also participate in biogeochemical cycles. For example, microorganisms are killed *via* viral infection and lysis, resulting in the release of nutrients and absorption by other microorganisms and plants, such as carbon, nitrogen, phosphorus, and sulfur (Kuzyakov and Mason-Jones, 2018). In marine systems, virus-driven carbon cycling is reported to account for 6%∼26% of the total carbon cycling (Weinbauer, 2004). Moreover, viruses indirectly impact biogeochemical cycles through abundant virus-encoded auxiliary metabolic genes (AMGs), including the genes related to the carbon (Hurwitz et al., 2013; Jin et al., 2019), nitrogen (Roux et al., 2016; Ahlgren et al., 2019; Gazitua et al., 2021), sulfur (Roux et al., 2016; Kieft et al., 2021), and phosphorus (Zeng and Chisholm, 2012; Goldsmith et al., 2015) cycles, which are discovered in marine and terrestrial ecosystems.

The previous researches about viruses were mainly focused on marine ecosystems. Compared with marine systems [ranging from 1 to 100; (Dion et al., 2020)], studies have shown a more highly variable range of virus-to-bacteria ratio (VBR) in soil [ranging from 0.001 to 8,200; (Williamson et al., 2017)]. In addition, viral communities in terrestrial ecosystems showed significant environmental specificity. Fierer et al. (2007) had demonstrated that the soil viral communities were significantly different from that in other environments (marine sediment, human fecal samples, and seawater environments) *via* metagenomic analysis. Therefore, facing the huge number of soil viruses and important ecological functions, we urgently need to pay more attention to the studies of soil viruses.

So far, studies on soil viral ecology have involved many soil types, such as desert soil (Adriaenssens et al., 2015), glacier soil (Han et al., 2017b), thawing permafrost soil (Trubl et al., 2016, 2018, 2019), forest soil (Jin et al., 2019; Liang et al., 2021), mud volcanic soil (Yu et al., 2019), agricultural soil (Bi et al., 2021), and wetlands (Jackson and Jackson, 2008). It has been confirmed that the abundance, diversity, and reproductive strategy of viruses in soil are affected by multiple factors. Soil pH was the main environmental driver of the viral community structure in agricultural soils (Bi et al., 2021) and also affected the viral attachment to soil particles (Gerba, 1984; Loveland et al., 1996). Soil type was significantly correlated with viral abundance (Williamson et al., 2017). In addition to the factors mentioned, the site altitude (Adriaenssens et al., 2017), temperature (Wen et al., 2004), soil organic matter (Chariou et al., 2019), soil water content (SWC) (Straub et al., 1992, 1993; Jin and Flury, 2002), etc., also impacted virus–host interaction. Wu et al. (2021) have verified that extreme changes in soil moisture have a great influence on the composition, activity, and potential functions of both DNA and RNA soil viruses. When the SWC decreased to < 5%, the infection ability of phage was significantly weakened, and the activity of phage was completely lost due to water volatilization (Straub et al., 1992, 1993). There was a distinct positive correlation between viral abundance and the SWC (Williamson et al., 2005).

Climate change is subtly changing the soil environment, affecting the species composition, community structure, and function of soil organisms, but how it affects soil viruses is still poorly understood (Kaisermann et al., 2017; Dong et al., 2020). For instance, the changes of precipitation lead to shift soil moisture, influencing soil biotic and abiotic properties (Cook et al., 2014). As mentioned, the SWC affects viral activity. However, it is currently unknown how changes in precipitation shape the soil virosphere and influence viral diversity, abundances, function, and replication strategies (lytic/lysogenic lifecycles) (Emerson, 2019; Van Goethem et al., 2019). The Qinghai-Tibet Plateau, known as the “the third pole” of the Earth, is considered to be one of the most sensitive areas to human activities and climate change and has become a research hotspot (Chen et al., 2013; Tao et al., 2018; Che et al., 2019). Despite the ecological importance of the Qinghai-Tibet Plateau, our knowledge on its biodiversity is notably limited. While some studies have focused on soil microbial community composition and diversity, including bacteria (Shen et al., 2019), fungi (Che et al., 2019), and archaea (Shi et al., 2019), there have been few reports of soil viruses. Based on the above views, this paper mainly focuses on (i) the abundance of soil viruses and microbes in the Qinghai-Tibet Plateau under different precipitation gradients and its potential environmental drivers; (ii) the differences of virus community composition and predicted hosts under different precipitation gradients; and (iii) the potential role in the biogeochemical cycling of soil viruses in the Qinghai-Tibet Plateau.

## Materials and Methods

### Sample Collection and Soil Physicochemical Properties

**FIGURE 1:**
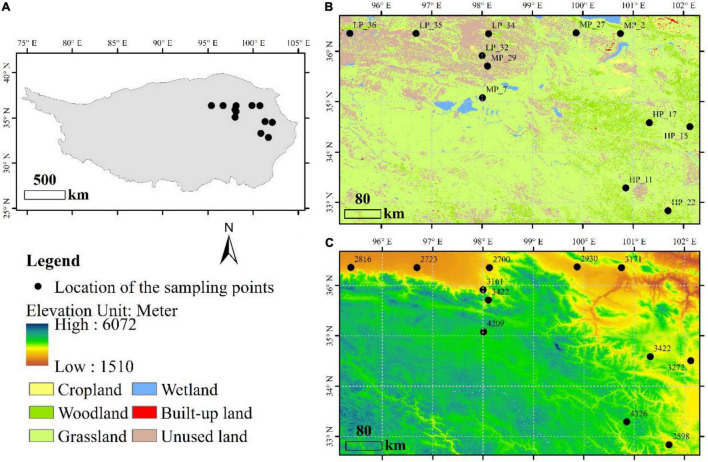
Distribution and description of sample sites, including geographical location **(A)**, land use type **(B)** and altitude **(C)**.

A total of 36 soil samples, located in the Qinghai Province of China, were selected from 12 field sites under three precipitation gradients (Figure 1). Samples in low precipitation (LP, mean annual precipitation < 200 mm) include LP_32, LP_34, LP_35, and LP_36. Samples in moderate precipitation (MP, mean annual precipitation is between 200 and 400 mm) include MP_2, MP_7, MP_27, and MP_29. Samples in high precipitation (HP, mean annual precipitation > 400 mm) include HP_11, HP_15, HP_17, and HP_22. All samples were from grasslands (Figure 1B) with the detailed information in Supplementary Table 1. Three adjacent samples were randomly taken from each site by inserting the coring device 10 cm into the soil surface. The coring device was cleaned with ethanol (70%, v/v) between sites to avoid cross sample contamination. Each of the soil samples was sieved through a 2 mm sieve to remove rocks and roots, mixed evenly, and placed into a clean zip-lock bag for bioinformatics and physicochemical analysis.

The soil physicochemical characteristics were determined according to established protocols (Ge et al., 2021; Shi et al., 2021). Briefly, soil pH was determined with a soil- to-water ratio of 1:2.5 (w/w) suspension using a pH meter (DELTA-320, China). Soil electrical conductivity (EC) was measured with a soil-to- water ratio of 1:5. SWC was determined by the oven-drying method at 105°C to constant weight. Soil organic matter (SOM) was estimated by the K_2_Cr_2_O_7_ oxidation-reduction colorimetric method. The TC and TN were determined by an elemental analyzer (Vario EL III-Elementar, Germany). NH_4_^+^-N and NO_3_^–^-N were extracted with 1 M KCl and measured by a continuous flow analyzer (SAN + +, Skalar, Holland). The soil’s total phosphorus (TP) was extracted by NaOH solution and determined by Mo-Sb colorimetric method (Wang et al., 2014).

### Epifluorescence Microscopy Enumeration

Virus-like particles (VLPs) and microbial abundances in each soil sample were estimated using epifluorescence microscopy (EFM) (Danovaro and Serresi, 2000; Thurber et al., 2009; Han et al., 2017a). 10 ml of 0.22 μm filtered amended 1% potassium citrate (AKC) buffer [1% potassium citrate resuspension buffer amended with 10% phosphate buffered-saline (PBS) and 150 mM magnesium sulfate (MgSO_4_)] was added to 3 ± 0.5 g soil (Trubl et al., 2016). Viruses and microbes were physically dispersed *via* 15 min of shaking at 150 rpm at room temperature. The supernatant-contained viruses and microbes were collected to new tubes. The resuspension steps above were repeated two more times to obtain approximately 30 ml supernatants and then filtered through a 0.45 μm Millex filter to remove large particles. The moderate filtrate was disposed with 5 U/ml DNase I (Thermo Fisher Scientific, Lithuania, European Union) and incubated at 37°C for 1 h to remove free DNA, then filtered through a 0.02 μm Anodisc Al_2_O_3_ filter membrane (25 mm diameter, Whatman; GE Healthcare, Kent, United Kingdom) supported by a 0.45 μm filter. The dried Anodisc filter membrane was stained with SYBR Green I (Invitrogen, Eugene, OR, United States) working solution (1:400) for 20 min in darkness at room temperature. The stained filter membrane was then mounted on a glass slide with ab antifade solution [50% glycerol, 50% phosphate-buffered saline (0.05 M Na_2_HPO_4_, 0.85% NaCl; pH 7.5), 0.1% p-phenylenediamine]. Subsequently, ten fields of view were randomly selected for observation under EFM (Nikon, Melville, NY, United States), and the abundances of VLPs and microbial cells and VMR were calculated.

### DNA Extraction, Library Construction, and Metagenomic Sequencing

The total DNA was extracted from 0.25 g of soil with the PowerLyser PowerSoil DNA isolation kit (Qiagen, Hilden, Germany), following the manufacturer’s protocol. The DNA extract was fragmented to an average size of about 400 bp using Covaris M220 (Gene Company Limited, Shanghai, China) for paired-end library construction. A paired-end library was constructed using NEXTFLEX Rapid DNA-Seq (Bioo Scientific, Austin, TX, United States). Paired-end sequencing was performed on the Illumina NovaSeq platform (Illumina Inc., San Diego, CA, United States) at Majorbio Bio-Pharm Technology Co., Ltd. (Shanghai, China) using NovaSeq Reagent Kits according to the manufacturer’s instructions.^1^ The sequencing depth was 20 Gbp for each sample. The DNA content of LP_34 and LP_36 samples is too little to meet the requirements of library construction.

### Analysis of Metagenomes

#### Quality Control, Assembly, and Identification of Viral Operational Taxonomic Units

The raw reads of the 30 samples obtained from the Illumina NovaSeq platform were cleaned using the Fastp tool to remove adapters and filter low-quality reads (length < 50 bp or with a quality value < 20 or having N bases) (Chen et al., 2018), followed by *de novo* assembly into contigs ≥ 500 bp in length under default conditions using MEGAHIT (Li et al., 2015) and clustered with PSI-CDHIT (Huang et al., 2010). Then, VirSorter (Roux et al., 2015), VIBRANT (Kieft et al., 2020), and DeepVirFinder (Ren et al., 2020) were used to detect dsDNA viral contigs from each assembly (≥ 1,000 bp contigs). Only ≥ 10 kb contigs were retained according to Minimum Information about an Uncultivated Virus Genome (Roux et al., 2019). Based on the Discovery Environment 2.0,^2^ only contigs from VirSorter categories 1, 2, 4, and 5 (high confidence) were retained, and the combined phages in VIBRANT were considered viral. DeepVirFinder runs according to its Python script,^3^ and the contigs with scores > 0.9 and *p* < 0.05 were considered viral. The identified viral contigs were then compiled and clustered at 98% nucleotide identity using cd-hit-est software (Li and Godzik, 2006), totally producing 557 viral operational taxonomic units (vOTUs).

#### Taxonomy and Functional Annotation and Host Prediction

Clean reads were classified using Kraken2 (Wood et al., 2019) against the reference sequences of the National Center for Biotechnology Information (NCBI) database (RefSeq, accessed December 2021) to identify bacterial, archaeal, and viral reads, and the relative abundance of viruses was directly obtained from the classification results of Kraken 2. The CAZymes identified from 557 vOTUs were automated on the dbCAN2 meta server based on CAZyme family specific HMMER (E-value < 1e^–15^, coverage > 0.35), DIAMOND (E-value < 1e^–102^), and Hotpep (frequency > 2.6, hits > 6) together (Zhang et al., 2018). The information of integrase was selected from identified putative viral AMGs in 557 vOTUs by DRAM-v (Shaffer et al., 2020); only the results with viral_bitScore > 60 and viral_E-value < 1e^−5^ were retained. Similarly, putative hosts were predicted from 557 vOTUs as an input file using PHISDetector (default parameters), a web tool that detects diverse *in silico* phage–host interaction signals (Zhou et al., 2020).

#### Statistical Analyses of the Metagenomes

The difference analysis of VLPs and bacterial abundance under different precipitate-on gradients were completed by Statistical Product and Service Solutions (SPSS) Statistics 26. Random forest models (Breiman, 2001) used to evaluate the relative importance of various factors influencing VLP abundance were performed by the “randomForest” and “rfPermute” packages on the R platform. LEfSe (linear discriminant analysis effect size) analysis was performed based on *p* < 0.05 and an LDA score > 2.0.^4^ Virus–host prediction results were generated manually by the Adobe Illustrator CS6.

### Data Availability

Metagenome read data are available in the NCBI Short Read Archive under BioProject ID PRJNA782356.

## Results

### Virus-Like Particles and Microbial Abundance and Environmental Driving Factors

Viruses and microbes in soil were enumerated using EFM. The abundance of virus-like particles (VLPs) ranged from 2.0 × 10^7^ (LP_36) to 1.0 × 10^10^ (HP_22) per gram of dry soil, and microbial abundance ranged from 1.0 × 10^8^ (MP_29) to 8.2 × 10^8^ (HP_15) per gram of dry soil. The virus-to-microbe ratio (VMR) also varied in different soils, ranging from 0.11 to 98.3 (Table 1). There were significant differences in VLP abundance among different precipitation gradients. The VLP abundance under HP was the highest, followed by MP and LP. Furthermore, the microbial abundance under HP was significantly higher than that under MP and LP. Interestingly, the VMR under HP was significantly higher than that under LP (Table 1).

**TABLE 1:** Virus-like particles (VLPs) and microbial abundance under different precipitation gradients.

Annual precipitation	Site	VLP abundance × 10^9^ gdw^–1^		Microbial abundance × 10^8^ gdw^–1^		Virus-to-microbe ratio (VMR)	
< 200 mm	LP_32	1.66 ± 0.50 a	A	1.3 ± 0.32 a	A	12.09 ± 0.96 abc	A
	LP_34	0.83 ± 0.61 a		2.9 ± 0.61 abc		2.39 ± 1.43 ab	
	LP_35	0.2 ± 0.02 a		1.8 ± 0.19 ab		1.12 ± 0.18 a	
	LP_36	0.02 ± 0.00 a		1.4 ± 0.16 a		0.11 ± 0.02 a	
200–400 mm	MP_2	1.21 ± 0.37 a	B	3.3 ± 1.28 abc	A	7.29 ± 4.72 ab	AB
	MP_7	4.29 ± 0.21 a		4.4 ± 1.71 abc		13.61 ± 5.15 abc	
	MP_27	0.23 ± 0.03 a		3.7 ± 0.57 abc		0.63 ± 0.03 a	
	MP_29	9.21 ± 3.59 b		1.0 ± 0.49 a		98.3 ± 13.00 d	
> 400 mm	HP_11	9.82 ± 2.22 b	C	5.4 ± 1.01 bcd	B	18.14 ± 1.76 bc	B
	HP_15	9.76 ± 1.21 b		8.2 ± 2.77 d		13.57 ± 2.45 abc	
	HP_17	9.68 ± 0.83 b		5.7 ± 0.91 cd		18.52 ± 5.10 bc	
	HP_22	10.02 ± 2.61 b		3.7 ± 0.27 abc		26.42 ± 5.77 c	

*Statistical differences within and between groups were determined using one-way ANOVA based on Duncan test, and all groups were labeled accordingly (i.e., a, b, c, A, B, and C; p < 0.05).*

**FIGURE 2:**
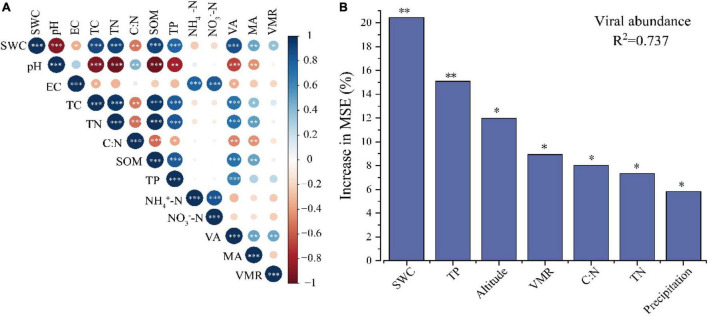
Environmental factors influencing virus-like particles (VLPs) abundance. Correlation among VLPs abundance, microbial abundance, VMR and soil physical chemical properties **(A)**. Random forest model evaluating the relative importance of various factors influencing VLP abundance **(B)**. Only the factors with significant influence are shown in the figure (top 7), *p < 0.05, ^**^p < 0.01.

Pearson correlations were used to evaluate the factors that might influence the soil VLP abundance, microbial abundance, and VMR. Across all samples, soil VLPs have a significant positive correlation with microbial abundance (*r* = 0.491, *p* < 0.01) (Figure 2A). The results showed significant positive correlations of VLPs and microbe counts with SWC (virus, *r* = 0.868, *p* < 0.001; microbe, *r* = 0.487, *p* < 0.01), total carbon (TC; virus, *r* = 0.701, *p* < 0.001; microbe, *r* = 0.399, *p* < 0.05), total nitrogen (TN; virus, *r* = 0.711, *p* < 0.001; microbe, *r* = 0.514, *p* < 0.01), soil organic matter (SOM; virus, *r* = 0.715, *p* < 0.001; microbe, *r* = 0.500, *p* < 0.01) and TP (virus, *r* = 0.652, *p* < 0.001; microbe, *r* = 0.500, *p* > 0.05). A significant negative correlation of VLPs was observed with C/N (*r* = −0.473, *p* < 0.01), soil pH (*r* = −0.677, *p* < 0.001), and EC (*r* = −0.350, *p* < 0.05). A significant negative correlation of microbial abundance was observed with C/N (*r* = −0.434, *p* < 0.01) and soil pH (*r* = −0.445, *p* < 0.01). Particularly, VMR was positively correlated with SWC (*r* = 0.371, *p* < 0.05) and VLP abundance (*r* = 0.484, *p* < 0.01). Furthermore, the random forest model was used to evaluate the relative importance of soil physical–chemical properties, biological factors, climate factors, and geographical location for predicting VLP abundance, which indicated that SWC was the main environmental factor affecting VLP abundance, followed by TP (*p* < 0.05) (Figure 2B).

### Viral Community Composition Under Different Precipitation Gradients

**FIGURE 3:**
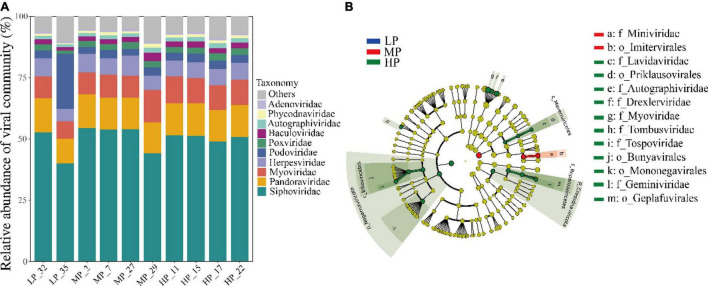
Taxonomic composition of soil viruses in the Tibetan Plateau, assessed for all virus-associated reads at the family level **(A)**. Only the top ten viral families were shown. Viral biomarkers in different precipitation based on LEfSe analysis **(B)**. Different colors represent different precipitation gradients and the circles from inside to outside correspond to kingdom to family.

A total of 54 viral orders, 152 families, and 362 genera were identified from the metagenomic data of 30 soil samples. At the family level, the average relative abundance of *Siphoviridae* (40.1%–54.5%) belonging to the order *Caudovirales* was the highest in all samples. The family *Pandoraviridae* (10.1%–13.9%), *Myoviridae* (7.2%–13.1%), and *Herpesviridae* (5.0%–8.2%) also accounted for a large proportion (Figure 3A). The relative abundance of *Podoviridae* (22.6%) in LP_35 was 5–7 times higher than that in other samples (2.7%–3.6%). Finally, other viral families like *Poxviridae*, *Baculoviridae*, *Autographiviridae*, *Phycodnaviridae*, and *Adenoviridae* accounting for a small proportion were also detected in each soil. The distribution of dominant viral families was greatly similar among most samples under three precipitation gradients. LEfSe analysis identified five orders and eight families of soil viruses that showed significantly different abundance among three precipitation gradients (Figure 3B). A total of 70.1% belonged to *Caudovirales*, and most of these belonged to *Siphoviridae* (50.2%). Only *Imitervirales* and *Miniviridae* were significantly enriched in MP. Four orders (*Priklausovirales*, *Bunyavirales*, *Mononegavirales*, and *Geplafuvirales*) and seven families were significantly enriched in HP.

### Differences Between Virus and Host Under Three Precipitation Gradients

**FIGURE 4:**
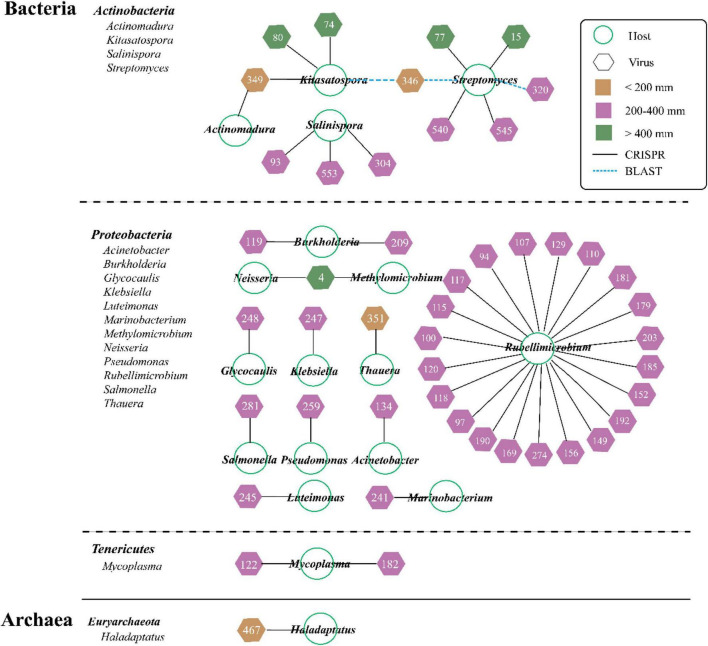
Predicted viral-host linkages under three precipitation gradients. About 47 vOTUs are linked to 4 host lineages by multiple lines of evidence, and the three prediction methods are represented by the different color-coded lines. Node shape denotes organism (circle for microbe and hexagon for virus), and number represents vOTU (Supplementary Table 2). Viral shapes are color-coded by three precipitation gradients (yellow for LP, purple for moderate precipitation, and green for high precipitation).

The predicted hosts including bacteria (primarily) and archaea were identified from 8.44% of vOTUs (47 out of 557 vOTUs) *via* the PHISDetector tool (Figure 4 ad Supplementary Table 2). Most of the individual links (47) occurred *via* CRISPRs (black solid line), with three *via* BLAST (blue dashed line). These hosts spanned 18 genera among four phyla (Bacteria: *Actinobacteria*, *Proteobacteria*, and *Tenericutes*; Archaea: *Euryarchaeota*). Most viruses were linked to only one host at the genus level; only three viruses were, respectively, linked to two hosts but always within the same order (vOTU_4, vOTU_346, and vOTU_349). Of all the hosts, *Rubellimicrobium* (involving 21 vOTUs) has the most association with viruses, followed by *Streptomyces* (involving 6 vOTUs). The overall number of virus–host linkages (pairs) under MP (38) was significantly higher than that under LP (6) and HP (6).

### Abundance and Diversity of Putative Auxiliary Carbohydrate Metabolic Genes in Soil Viruses

**FIGURE 5:**
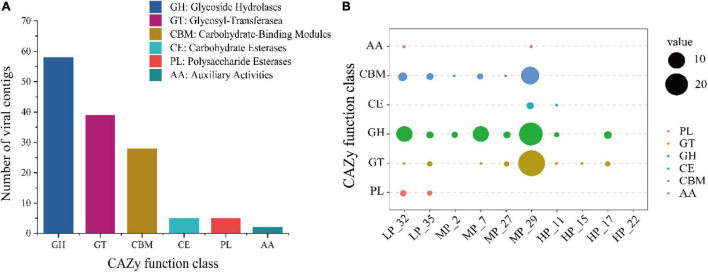
Abundant auxiliary CAZyme from surface soil viruses in the Tibetan Plateau. Annotation of viral carbohydrate metabolism-related contigs (> 10 kb) in the CAZy database **(A)**. The distribution of viral auxiliary CAZyme in each sample **(B)**.

To clarify the viral role in carbon cycling in soils, 557 vOTUs were further annotated for carbohydrate-active enzymes (CAZymes) by the dbCAN server based on the recognition of the CAZyme signature domain. According to results, 23.0% of vOTUs (128 of 557 vOTUs) carried 137 CAZyme genes (Figure 5A). These genes belonged to six CAZyme functional classes, most of which are affiliated to glycoside hydrolases (GHs, 42.3% of 137 viral CAZymes genes), followed by glycosyl-transferase (GTs, 28.5%) and carbohydrate-binding modules (CBMs, 20.4%) (Figure 5A). The most annotated CAZymes genes were found in soil viruses under MP, especially MP_29, followed by LP. However, the soil viruses under HP carried fewer CAZyme genes, only involving CE, GH, and GT (Figure 5B). CBM50 and GH23 were the most widely distributed, with different distributions in five samples (Supplementary Figure 1).

## Discussion

### Soil Water Content Was the Main Driving Factor for Viral Abundance on the Qinghai-Tibet Plateau

It has been confirmed that the abundance, diversity, and reproductive strategy of viruses in soil are affected by multiple factors, such as soil types, physical and chemical properties, cover plants, and so on (Williamson et al., 2017; Liang et al., 2021). In order to expand our understanding of the distribution of soil viruses across different precipitation gradients, the abundance of microorganisms and viruses in 36 soil samples across three precipitation gradients was quantified by fluorescence microscopy. This method only quantified free and adsorbed virus particles in soils, ignoring temperate viruses, so the true abundance of soil viruses was underestimated. Our data showed that there were significant differences in both VLPs and the microbial abundance among different precipitation gradients; both of them increased with precipitation and reached the highest at HP (Table 1), which was consistent with previous studies that soil tends to have more abundant viruses and bacteria under the condition of high moisture content and organic matter (Srinivasiah et al., 2008). In our data, high carbon and nitrogen nutrients in wet soil are beneficial to the growth of microorganisms (Supplementary Table 3), which may make it more conducive for the encounter of a virus and host, resulting in more viruses. Srinivasiah et al. (2008) found that bacterial abundance increased by 84-fold after the addition of C- and N-rich substrates. Also, VLP counts were highly correlated with multiple soil physical and chemical factors compared with microbial abundance, such as SWC, TN, TC, SOM, TP, pH, EC, and C/N (Figure 2A); the result of the random forest showed that SWC was the main environmental factor affecting VLP abundance (*p* < 0.05) (Figure 2B). Therefore, we supposed that viruses were more sensitive to environmental factors than microbes and SWC was a key environmental factor driving VLP abundance in soil, which indirectly reflected the impact of climate change (average annual precipitation) on VLP abundance and provided a theoretical basis for the conjecture of Williamson et al. (2017) who strongly suspected that the SWC was a key environmental parameter driving both bacterial and viral abundance in soils.

In this study, the VMR, an index to measure viral activity and study the virus–host interaction in the environment (Ogunseitan et al., 1990; Wommack and Colwell, 2000; Parikka et al., 2017), fluctuated over four orders of magnitude (from 0.11 to 98.3) and was significantly higher in HP than that in LP (Table 1). Since VMR is the ratio of VLPs to microbial abundance, its value has to do with the factors controlling both VLPs and microbial abundance. As previous results showed, both VLPs and microbial abundance have a significant positive correlation with SWC, and SWC mainly contributed to the variability in VLP abundance (Figure 2). In addition, soil VLPs have a significantly positive correlation with microbial abundance (*r* = 0.491, *p* < 0.01) (Figure 2A),as has been observed in other soils (Williamson et al., 2005; Liang et al., 2019a,^2020^). With regard to the correlations between viral abundance and replication strategy and microbial abundance, several hypothesized models have been previously reported. The KtW model proposed that viral predation was density and frequency dependent, while lysogeny is facilitated at low host density (Thingstad and Lignell, 1997). Moreover, a study on coral reef samples has found that both viral abundance and VMR were reduced at high host density and confirmed that lysogeny is more prevalent in an environment with high host densities. The “PtW” model was thus presented (Knowles et al., 2016). Based on our results, the viral lytic efficiency was higher in soils with higher water content, and the dynamic changes between viruses and hosts were more consistent with the KtW model (Thingstad and Lignell, 1997). While the sampling pool of this work was limited, this study indicates the interesting results of viral distribution and replication strategies likely being influenced by SWC and sheds light on the drivers of viral community dynamics in the complicated soil environment. Future effort is needed to reveal the relationship of soil properties and other factors with viral distribution and production and, in turn, the role of viruses in biogeochemical cycling in the soil of the Qinghai-Tibet Plateau.

### Viral Diversity and Host Prediction Across Three Precipitation Gradients

Taxonomic diversity analysis revealed that *Caudovirales* (70.1%) was the major viral group in soil. The relative abundance of the *Siphoviridae* family was the highest in all samples (Figure 3A). This result is consistent with that in southeastern United States agricultural soil and Antarctic soil (Adriaenssens et al., 2017; Liang et al., 2019a). Many previous studies have shown that *Siphoviridae* was the dominant viral family in both soil and marine systems (Jin et al., 2019; Gao et al., 2020; Zheng et al., 2021). The family *Pandoraviridae*, *Myoviridae*, and *Herpesviridae* also accounted for a large proportion. Pandoravirus, the second largest giant virus after Mimivirus, can infect amoeba. Its DNA genomes can reach 2.5 Mb, much larger than that of other viruses (Philippe et al., 2013). Pandoraviruses were rarely detected in soil habitats; however, a most recent study by Legendre et al. (2018) found that *Pandoravirus quercus* can be isolated from ground soil in Marseille (France). In our results, *Pandoraviridae* family accounted for 10.1%–13.9% in each soil sample, which may provide convenient conditions for studying the diversity and evolution of *Pandoraviridae* family in soil. Importantly, the distribution of dominant viral families (top 10) was greatly similar among most samples under three precipitation gradients, indicating that the change of precipitation had little influence on the composition of soil viruses. While the viral species composition at a family level was similar among samples, different dominant virus populations were significantly enriched in soil with MP and HP as the LEfSe analysis result showed, respectively. For example, the families *Imitervirales* and *Miniviridae* were significantly enriched in soil under MP (Figure 3B). However, these results are highly dependent on the viral database that is still much incomplete, which will be further refined, as more environmental viruses are discovered and the database is improved.

In order to examine these viruses’ impacts on the microbial communities and processes, we sought to link them to their hosts *via* the PHISDetector tool (Zhou et al., 2020). In this study, only 8.44% of vOTUs has been assigned to hosts including bacteria and archaea that spanned 18 genera among four phyla (Bacteria: *Actinobacteria*, *Proteobacteria*, and *Tenericutes*; Archaea: *Euryarchaeota*) (Figure 4). Consistent with previous reports, the majority of hosts were annotated as bacteria. It was reported that both *Proteobacteria* and *Actinobacteria* were the dominant soil bacteria taxa (Liang et al., 2021; Wu et al., 2021; Zheng et al., 2021). In addition, compared with grassland soils in Kansas (Wu et al., 2021) and samples from Lake Michigan (Malki et al., 2015), the host range was narrow because most viruses were linked to only one host at the genus level, which was similar to the findings of Trubl et al. (2018) and ter Horst et al. (2021). Particularly, the overall number of virus–host linkages (pairs) in MP (38) was significantly higher than that in LP (6) and HP (6). The site-specific vOTUs had different predicted hosts, thus illustrating the unique assemblages of soil viruses and hosts across sites with differences in precipitation. Interestingly, only vOTU_467 from LP_35 sample located in the Qaidam Basin was assigned to archaea, probably resulting from the highest salt concentration (Supplementary Table 3). The majority of archaea phages originated in thermophilic or extremely halophilic environments (Mochizuki et al., 2010). The Qaidam Basin was originally a huge lagoon. Due to the continuous elevation of the Qinghai-Tibet Plateau, reduction of rainfall, and evaporation of water, this huge natural salty lake basin was formed. Overall, further studies of viral communities and the virus–host interaction can fuel our knowledge of viral ecology in different soil matrices and how biotic/abiotic factors play a role in structuring viral communities.

### Viruses May Play a Potential Role in Soil Carbon Cycling on the Qinghai-Tibet Plateau

Soil is the largest carbon pool in terrestrial ecosystems (Post et al., 1982), and the Tibetan Plateau stores the most abundant soil organic carbon (Stockmann et al., 2015; Liang et al., 2019b). In the past, many studies focused on the influence of microbes on the soil carbon cycle, including bacteria and fungi, but ignored the contribution of viruses. It was confirmed that viruses affect ecosystem carbon processing *via* the controls of top–down (lysing dominant microbial hosts) and bottom–up (carrying AMGs) (Brum and Sullivan, 2015; Trubl et al., 2018). Virus-encoded diverse AMGs could enhance or expand the host metabolic pathways, thus opening up new ecological niches and affecting biogeochemistry (Roux et al., 2016; Gazitua et al., 2021). For example, photosystem I and II genes were obtained by the marine cyanophages from cyanobacteria, and the expression of these genes during infection promoted the photosynthetic output of host cells (Thompson et al., 2011). Thus, to reveal the potential contribution of viruses to the carbon cycle on the Tibetan Plateau, potential CAZymes in the soil viral genomes were annotated. Finally, 22.2% of vOTUs (128 vOTUs) carrying 59 CAZyme genes (categories) that spanned 137 CAZyme genes were further annotated, with most of CAZymes affiliated to polysaccharide hydrolase activities, implying that viruses may play a potential role in the decomposition of organic carbon on the Qinghai-Tibet Plateau (Figure 5A). However, compared with farmlands (10 CAZymes genes) (Bi et al., 2021) and mangroves (27 CAZyme genes) (Jin et al., 2019), soil viruses in the Qinghai-Tibet Plateau carried more diverse CAZyme genes (species), such as GH5, GH8, CE11, CE14, and so on, indicating the higher occurrence of CAZymes and non-negligible roles of viruses in the soil carbon cycle. This difference might result from the environmental specificity of CAZymes (Bi et al., 2021). Our data suggested that the GHs responsible for the breakdown of complex organic matter were the most abundant, supporting previous findings (Emerson et al., 2018). Further, we found that CAZyme genes were the most abundance in samples under MP (Figure 5B), especially in MP_29, which corresponded to the result of host prediction. However, there were the highest VLPs abundance and the lowest CAZyme genes under HP, probably resulting from virus-carrying AMGs that mainly exist in the lysogenic state. The distribution of integrase genes also supported high abundant lysogenic viruses presented in MP (Supplementary Figure 2 and Supplementary Table 4). In addition, we also identified more specific auxiliary carbohydrate metabolism genes, including GHs, glycosyl transferases, polysaccharide lyases, carbohydrate esterases, and carbohydrate-binding modules (Supplementary Figure 1), implying that more ecological functions of viruses are yet to be discovered. Together, these results suggest that viral infections contribute to soil ecosystem functioning and that further interrogation of soil viral communities will yield a more comprehensive understanding of complex functional networks and ecosystem processes in soil.

## Conclusion

This study provides a new knowledge of the abundance, composition and host diversity, and potential biogeochemical impacts of soil viruses in the grassland soil of Qinghai-Tibet Plateau across three precipitation gradients. VLP abundance is positively correlated with microbial abundance; both of them reach the highest in wet soil, and the SWC mainly contributes to the variability in VLP abundance. In addition, based on LEfSe analysis, we find that different dominant virus populations were significantly enriched in soil with MP and HP. Interestingly, Pandoraviruses, the second largest giant virus after Mimivirus, are detected abundantly in soil. High host diversity and abundant CAZyme genes were shown in soils with moderate precipitation. Finally, abundant CAZymes represented by GHs were identified in our study, indicating that soil viruses may play a potential role in the carbon cycle on the Qinghai-Tibet Plateau, and some novel auxiliary carbohydrate metabolism genes were also identified. Overall, the results of this study indicate major differences in soil viruses along the precipitation gradient and provide a theoretical basis for the influence of climate change on the soil virosphere.

## Data Availability Statement

The datasets presented in this study can be found in online repositories. The names of the repository/repositories and accession number(s) can be found in the article/Supplementary Material.

## Author Contributions

L-MZ, J-ZH, and L-LH designed the research. L-MZ, S-YL, H-YW, and YG collected the soil samples. M-MC, S-YL, and L-LH lead the laboratory work. M-MC, S-JC, and BH performed the data analysis. M-MC, LB, and L-LH interpreted the results and wrote the manuscript in close consultation from all authors. All authors contributed to the article and approved the submitted version.

## Conflict of Interest

The authors declare that the research was conducted in the absence of any commercial or financial relationships that could be construed as a potential conflict of interest. The handling editor declared a shared affiliation with one of the authors H-YW at the time of review.

## Publisher’s Note

All claims expressed in this article are solely those of the authors and do not necessarily represent those of their affiliated organizations, or those of the publisher, the editors and the reviewers. Any product that may be evaluated in this article, or claim that may be made by its manufacturer, is not guaranteed or endorsed by the publisher.

## Footnotes

1
www.illumina.com

2
https://de.cyverse.org

3
https://github.com/jessieren/DeepVirFinder

4
http://huttenhower.sph.harvard.edu/galaxy
