# Supplementary material for: Distribution Characteristics of Soil Viruses Under Different Precipitation Gradients on the Qinghai-Tibet Plateau
Source: Front Microbiol. 2022 Apr 7;13:848305. doi: 10.3389/fmicb.2022.848305 (PMC9022101; doi:10.3389/fmicb.2022.848305)
Supplement: Supplementary file 1 [file Table_1.docx]

**Figure S1** The distribution of viral auxiliary CAZyme genes in each sample, including 59 CAZyme genes.


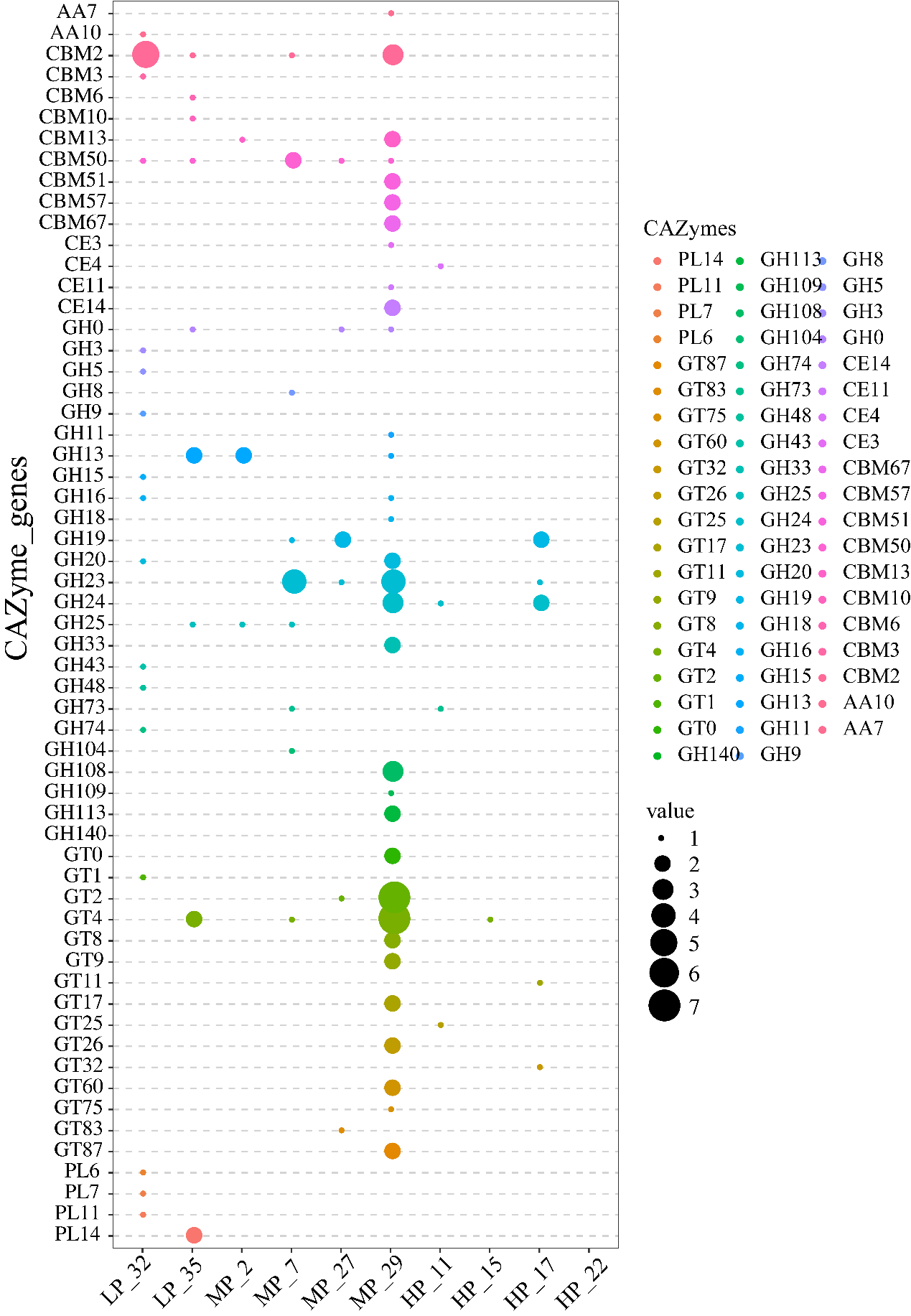


**Figure S2** The distribution **(A)** and maximum likelihood tree **(B)** of integrase genes carried by viruses under three precipitation gradients. The tree was bootstrapped with 1000 sub-replicates, and bootstrap scores > 50% are flagged with circles. The vOTU of soil across LP, MP and HP are coloured in yellow, blue and pink, respectively. **
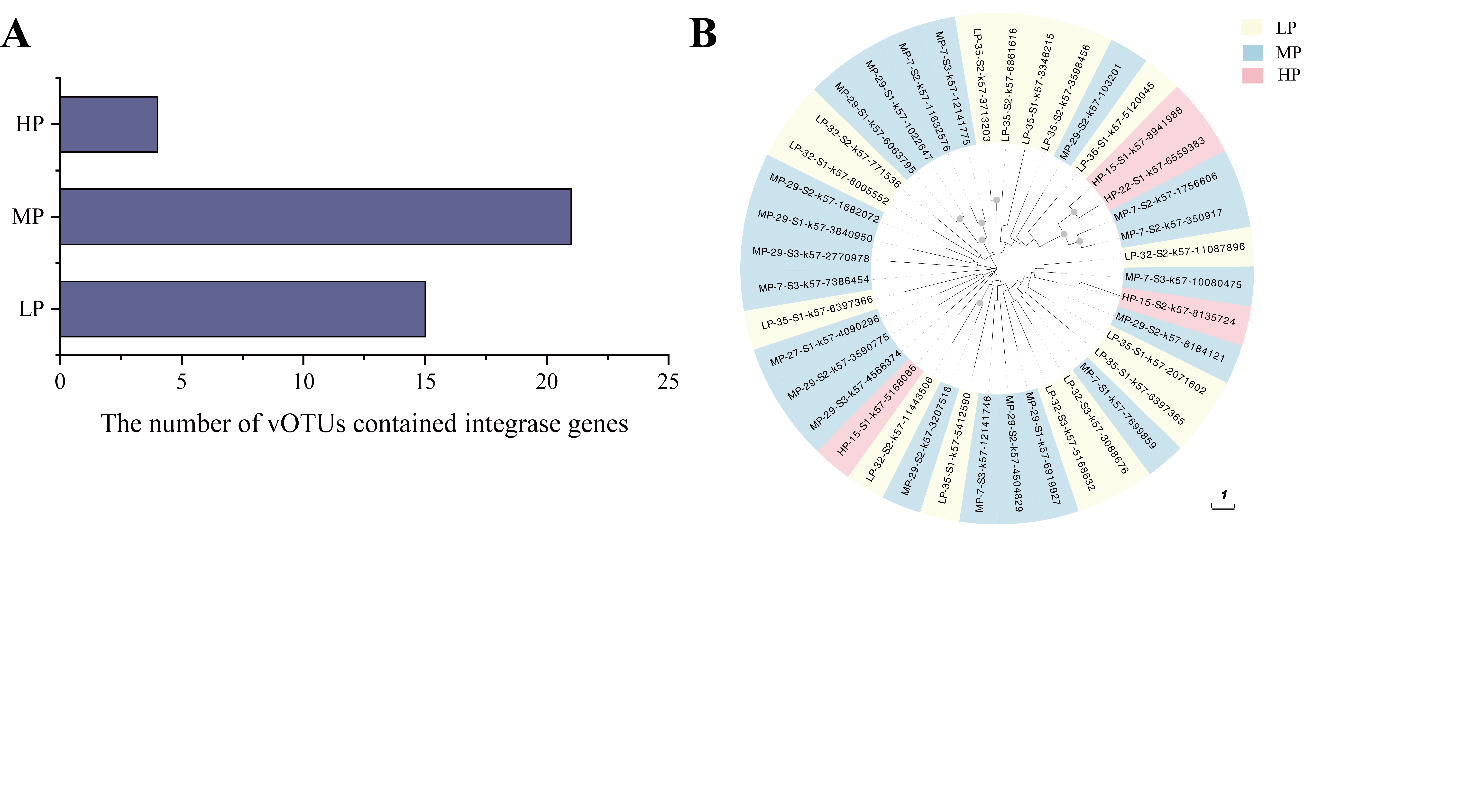
**

**Table S1** Sampling site location and description.

| **Sample** | **Longitude** | **Latitude** | **Annual**  **precipitation** | **Altitude**  **/m** | **Land use** |
| --- | --- | --- | --- | --- | --- |
| LP_32 | 98°00′01.581″ | 35°54′47.209″ | <200 mm | 3161 | Grassland |
| LP_34 | 97°05′3758.789″ | 36°21′02.604″ | <200 mm | 2700 | Grassland |
| LP_35 | 96°41′13.691″ | 36°21′12.862″ | <200 mm | 2723 | Grassland |
| LP_36 | 95°22′28.430″ | 36°21′18.926″ | <200 mm | 2816 | Grassland |
| MP_2 | 100°44′49.69″ | 36°21′5.22″ | 200-400 mm | 3171 | Grassland |
| MP_7 | 98°00′33.062″ | 35°4′39.216″ | 200-400 mm | 4209 | Grassland |
| MP_27 | 99°52′00.744″ | 36°21′57.728″ | 200-400 mm | 2930 | Grassland |
| MP_29 | 98°06′22.870″ | 35°42′27.180″ | 200-400 mm | 3422 | Grassland |
| HP_11 | 100°51′08.621″ | 33°17′26.637″ | >400 mm | 4326 | Grassland |
| HP_15 | 102°07′26.130″ | 34°30′11.623″ | >400 mm | 3272 | Grassland |
| HP_17 | 101°19′10.096″ | 34°34′58.982″ | >400 mm | 3422 | Grassland |
| HP_22 | 101°41′29.806″ | 32°50′10.422″ | >400 mm | 3598 | Grassland |

**Table S2** Prediction of viral-host linkages in soil under three precipitation gradients.

| Annual precipitation | Sample | Viral contig  number | Length /kb | Host_id | Host_genus | Predict_signal | Overall_score |
| --- | --- | --- | --- | --- | --- | --- | --- |
| < 200 mm | LP_32 | 349 | 46314 | NZ_BCQS01000026; NZ_JNYE01000089 | *Actinomadura*; *Kitasatospora* | crispr | 1 |
|  |  | 346 | 10193 | NZ_CP020567; CP023407; CP029254; NZ_CP016825; NZ_CP015726; NZ_CP021744; NZ_CP010519; CP029601; NZ_CP023992; NZ_LT963352; CP031455; LT962942; NZ_CP031455; CP033073; CP033071; LT963352; NZ_CP029254; NZ_LT962942; CP034587 | *Kitasatospora*; *Streptomyces* | blast | 1 |
|  |  | 351 | 10874 | NZ_AMXA01000002 | *Thauera* | crispr | 1 |
|  | LP_35 | 467 | 19500 | NZ_JDTH01000008 | *Haladaptatus* | crispr | 1 |
| 200-400 mm | MP_2 | 320 | 11481 | NC_013929 | *Streptomyces* | blast | 1 |
|  | MP_7 | 553 | 12807 | NZ_KI911420; NZ_AZVW01000049 | *Salinispora* | crispr | 1 |
|  |  | 540 | 11060 | NZ_FR873693 | *Streptomyces* | crispr | 1 |
|  |  | 545 | 10185 | NZ_LFML01000079 | *Streptomyces* | crispr | 1 |
|  | MP_27 | 93 | 64485 | NZ_AZWY01000006 | *Salinispora* | crispr | 1 |
|  | MP_29 | 134 | 41823 | CP030031; NZ_CP030031 | *Acinetobacter* | crispr | 1 |
|  |  | 119 | 10656 | NZ_CP009798 | *Burkholderia* | crispr | 1 |
|  |  | 209 | 10656 | NZ_CP009798 | *Burkholderia* | crispr | 1 |
|  |  | 248 | 18076 | CP018911 | *Glycocaulis* | prophage | 1 |
|  |  | 245 | 27658 | NZ_JAAN01000005 | *Luteimonas* | crispr | 1 |
|  |  | 241 | 53906 | NZ_KB899093 | *Marinobacterium* | crispr | 1 |
|  |  | 122 | 106518 | NZ_CP007229; LR214971 | *Mycoplasma* | crispr | 1 |
|  |  | 182 | 165467 | LR214971; NZ_CP007229 | *Mycoplasma* | crispr | 1 |
|  |  | 259 | 11665 | LR214971 | *Mycoplasma* | crispr | 1 |
|  |  | 100 | 68474 | NZ_CP007229 | *Mycoplasma* | crispr | 1 |
|  |  | 120 | 38490 | LR214971 | *Mycoplasma* | crispr | 1 |
| Annual precipitation | Sample | Viral contig  number | Length /kb | Host_id | Host_genus | Predict_signal | Overall_score |
| 200-400 mm | MP_29 | 97 | 92794 | NZ_LZQK01000223 | *Pseudomonas* | crispr | 1 |
|  |  | 247 | 18112 | NZ_KQ089365; NZ_KQ089406; NZ_KQ089701; NZ_GL882852; NZ_CP011624; NZ_MPYK01000084; NZ_MPYL01000018; NZ_ARRZ01000040; NZ_JWRK01000001; NZ_JQDX01000015; NZ_JQDY01000013; NZ_LLWB01000118; NZ_JQCX01000013; NZ_LXNC01000004; NZ_AOPN02000190; NZ_JQCW01000004; NZ_JRRF01000004; NZ_FLJW01000005; NZ_LNHK01000027; NZ_LNHF01000038; NZ_LNHE01000037; NZ_FLIS01000011; NZ_FLDR01000006; NZ_LAFX01000003; NZ_LAFW01000003; NZ_JXBF01000003; NZ_CCGZ01000033; NZ_FLEP01000006; NZ_LAFV01000003;  NZ_FLIO01000002; NZ_FLIM01000003; NZ_LAFU01000002; NZ_CCHJ01000296; NZ_FLEK01000006; NZ_MPCF01000002; NZ_FLEL01000005; NZ_MNAI01000003; NZ_FLIH01000005; NZ_CCGX01000012; NZ_FLEM01000002; NZ_LJCD01000008; NZ_FLIE01000005; NZ_FBRA01000004; NZ_FLHA01000002; NZ_CCIL01000001; NZ_CCHH01000003; NZ_CCJA01000016; NZ_CCID01000021; NZ_CCGB01000034; CP025963; CP036442; CP029582; CP031795; CP023249; CP023839; CP032222; CP024429; CP019160; CP027042; CP024916; NZ_CP018695; NZ_CP019160; NZ_CP024834; NZ_CP014755; NZ_CP024429; NZ_CP012426; NZ_CP017934; NZ_CP017985; NZ_CP021757; NZ_CP023249; NZ_CP013322; NZ_CP025963; NZ_CP018719; NZ_CP024458; NZ_CP029582; NZ_CP021939; NZ_CP024916; NZ_CP024838; NZ_CP018707; NZ_CP018701; NZ_CP015025; NZ_CP021740; NZ_CP018713; NZ_CP023913; NZ_CP015500; NZ_CP021944; NZ_LN824133; NZ_CP018140; NZ_CP018686 | *Klebsiella* | crispr | 1 |
|  |  | 118 | 28560 | NZ_KE557322 | *Rubellimicrobium* | crispr | 1 |
|  |  | 115 | 37983 | NZ_KE557322 | *Rubellimicrobium* | crispr | 1 |
|  |  | 117 | 15722 | NZ_KE557322 | *Rubellimicrobium* | crispr | 1 |
|  |  | 94 | 95973 | NZ_KE557322 | *Rubellimicrobium* | crispr | 1 |
| Annual precipitation | Sample | Viral contig  number | Length /kb | Host_id | Host_genus | Predict_signal | Overall_score |
| 200-400 mm | MP_29 | 107 | 17806 | NZ_KE557322 | *Rubellimicrobium* | crispr | 1 |
|  |  | 129 | 62268 | NZ_KE557322 | *Rubellimicrobium* | crispr | 1 |
|  |  | 110 | 103350 | NZ_KE557322 | *Rubellimicrobium* | crispr | 1 |
|  |  | 181 | 20342 | NZ_KE557322 | *Rubellimicrobium* | crispr | 1 |
|  |  | 179 | 102212 | NZ_KE557322 | *Rubellimicrobium* | crispr | 1 |
|  |  | 203 | 14926 | NZ_KE557322 | *Rubellimicrobium* | crispr | 1 |
|  |  | 185 | 103327 | NZ_KE557322 | *Rubellimicrobium* | crispr | 1 |
|  |  | 152 | 62485 | NZ_KE557322 | *Rubellimicrobium* | crispr | 1 |
|  |  | 192 | 20935 | NZ_KE557322 | *Rubellimicrobium* | crispr | 1 |
| 1 |  | 149 | 13566 | NZ_KE557322 | *Rubellimicrobium* | crispr | 1 |
|  |  | 156 | 15716 | NZ_KE557322 | *Rubellimicrobium* | crispr | 1 |
|  |  | 169 | 38560 | NZ_KE557322 | *Rubellimicrobium* | crispr | 1 |
|  |  | 190 | 37980 | NZ_KE557322 | *Rubellimicrobium* | crispr | 1 |
|  |  | 274 | 34910 | NZ_KE557322 | *Rubellimicrobium* | crispr | 1 |
|  |  | 304 | 10534 | NZ_KE557322 | *Rubellimicrobium* | crispr | 1 |
|  |  | 281 | 14826 | NZ_KE557322 | *Rubellimicrobium* | crispr | 1 |
| > 400 mm | MP_11 | 4 | 45002 | NZ_CM001475; LR134533 | *Methylomicrobium*; *Neisseria* | crispr | 1 |
|  | MP_15 | 15 | 35224 | NZ_LIRL01000794 | *Streptomyces* | crispr | 1 |
|  | MP_17 | 30 | 34548 | NZ_CP015606 | Not know | crispr | 1 |
|  | MP_22 | 74 | 21854 | NZ_LBHA01000124; NZ_CP020568 | Kitasatospora | crispr | 1 |
|  |  | 80 | 15134 | NZ_KK853998 | Kitasatospora | crispr | 1 |
|  |  | 77 | 32919 | NZ_AZWL01000023 | Streptomyces | crispr | 1 |

**Table S3** Physical and chemical properties.

| Sample | LP_32 | LP_34 | LP_35 | LP_36 | MP_2 | MP_7 | MP_27 | MP_29 | HP_11 | HP_15 | HP_17 | HP_22 |
| --- | --- | --- | --- | --- | --- | --- | --- | --- | --- | --- | --- | --- |
| Average annual precipitation | < 200 mm | < 200 mm | < 200 mm | < 200 mm | 200-400 mm | 200-400 mm | 200-400 mm | 200-400 mm | > 400 mm | > 400 mm | > 400 mm | > 400 mm |
| SWC (%) | 6.9±0.5 | 12.8±1.1 | 1.4±0.3 | 1.0±0.1 | 11.8±0.2 | 9.8±0.1 | 2.7±0.5 | 29.5±1.2 | 49.8±0.6 | 41.7±1.9 | 39.1±1.4 | 55.8±4.4 |
| pH | 8.7±0.1 | 8.5±0.1 | 8.5±0.1 | 8.4±0.1 | 8.1±0.0 | 8.2±0.0 | 8.6±0.1 | 8.5±0.1 | 5.8±0.0 | 6.8±0.2 | 7.4±0.1 | 5.6±0.0 |
| EC (μS/cm) | 117±9 | 19093±  2100 | 39420±  16011 | 5485±  2624 | 169±8 | 122±5 | 73±6 | 2936±376 | 95±3 | 100±16 | 131±6 | 90±14 |
| TC (g/kg) | 22.0±1.5 | 20.0±1.0 | 15.9±1.6 | 13.1±0.6 | 31.4±0.2 | 21.1±0.1 | 11.1±0.3 | 21.9±1.4 | 51.0±1.4 | 33.9±2.3 | 34.1±1.2 | 61.2±3.5 |
| TN (g/kg) | 0.5±0.1 | 0.4±0.0 | 0.5±0.0 | 0.1±0.0 | 2.0±0.1 | 0.9±0.0 | 0.4±0.0 | 0.6±0.1 | 4.7±0.1 | 3.3±0.2 | 3.1±0.1 | 5.5±0.2 |
| C/N | 48.3±9.4 | 58.2±3.3 | 35.2±6.1 | 155.3±27.7 | 16.1±0.8 | 24.4±0.5 | 29.9±1.5 | 41.3±6.0 | 11.0±0.1 | 10.4±0.2 | 11.1±0.2 | 11.2±0.2 |
| SOM (g/kg) | 10.9±2.9 | 12.9±0.6 | 19.3±4.8 | 3.3±0.8 | 33.2±0.6 | 19.1±0.7 | 9.0±0.4 | 14.2±1.9 | 87.3±1.8 | 59.1±3.9 | 58.6±2.1 | 104.0±5.5 |
| TP (g/kg) | 6.1±0.1 | 0.4±0.0 | 5.5±0.2 | 4.8±0.2 | 5.8±0.1 | 3.4±0.0 | 2.7±0.1 | 6.0±0.1 | 10.2±0.1 | 7.8±0.1 | 6.9±0.1 | 10.3±0.1 |
| NH_4_^+^-N (mg/kg) | 14.7±0.6 | 14.1±0.9 | 50.0±12.7 | 18.9±3.0 | 16.1±2.0 | 11.4±1.0 | 11.6±2.8 | 10.4±3.3 | 13.7±0.8 | 17.4±1.3 | 8.4±1.4 | 19.3±2.1 |
| NO_3_^-^-N (mg/kg) | 17.8±3.9 | 78.4±33.1 | 721.6±313.3 | 325.6±214.8 | 79.9±42.7 | 84.4±20.6 | 21.1±6.1 | 22.5±15.4 | 179.1±13.6 | 93.0±2.8 | 93.9±10.5 | 170.0±22.3 |

**TABLE S4** Integrase gene annotation of metagenome.

| vOTU | Kegg_id | Kegg_hit | Viral_id | Viral_hit | Pfam_hits | Vogdb_description |
| --- | --- | --- | --- | --- | --- | --- |
| 15_S1_k57_5168085_flag_0_multi_55.5672_len_44718 | K14059 | integrase | NP_852561.1 | NP_852561.1 DNA integration/  recombination/invertion protein  [Bacillus phage phBC6A52] | Phage integrase family [PF00589.24] | sp\|P22877\|INTR_SACER Integrase; XhXr |
| 15_S1_k57_8941988_flag_0_multi_15.3233_len_24344 | K06400 | site-specific  DNA recombinase | YP_009950750.1 | YP_009950750.1 serine integrase  [Mycobacterium phage Leston] | Resolvase, N terminal domain  [PF00239.23]; Recombinase  [PF07508.15] | sp\|P03014\|PINE_ECOLI Serine recombinase PinE; Xr |
| 15_S2_k57_8135724_flag_3_multi_12.9942_len_47262 | K04763 | integrase/recombinase XerD | YP_009196981.1 | YP_009196981.1 integrase/Rec-ombinase [Bacillus phage Stills] | Phage integrase family [PF00589.24] | sp\|P22877\|INTR_SACER Integrase; XhXr |
| 22_S1_k57_6559383_flag_1_multi_11.5133_len_30737 | K06400 | site-specific DNA recombinase | YP_009950844.1 | YP_009950844.1 serine integrase  [Mycobacterium phage Paola] | Resolvase, N terminal domain  [PF00239.23]; Recombinase  [PF07508.15] | sp\|P03014\|PINE_ECOLI Serine recombinase PinE; Xr |
| 27_S1_k57_4090296_flag_0_multi_12_8346_len_10309-cat_2 | K14059 | integrase | NP_852561.1 | NP_852561.1 DNA integration/re-combination/invertion protein  [Bacillus phage phBC6A52] | Phage integrase family [PF00589.24] | sp\|P22877\|INTR_SACER Integrase; XhXr |
| 29_S1_k57_3840950_flag_0_multi_12_8658_len_41823-cat_2 |  |  |  |  | Phage integrase family [PF00589.24]; Arm DNA-binding domain  [PF13356.8] | sp\|P22877\|INTR_SACER Integrase; XhXr |
| 29_S1_k57_6063795_flag_1_multi_11_0000_len_61403-cat_2 |  |  | YP_007237580.1 | YP_007237580.1 site-specific  Integrase [Cronobacter phage  ENT47670] | Phage integrase family [PF00589.24]; Phage integrase, N-terminal SAM-like domain [PF02899.19]; Phage  integrase, N-terminal SAM-like  domain [PF13495.8] | sp\|P22877\|INTR_SACER Integrase; XhXr |
| vOTU | Kegg_id | Kegg_hit | Viral_id | Viral_hit | Pfam_hits | Vogdb_description |
| 29_S1_k57_6919927_flag_1_multi_7.0876_len_24440 |  |  |  |  | Phage integrase family [PF00589.24]; Phage integrase, N-terminal SAM-like domain [PF02899.19] | sp\|P22877\|INTR_SACER Integrase; XhXr |
| 29_S2_k57_1022647_flag_0_multi_13.6825_len_38148 |  |  | YP_007237580.1 | YP_007237580.1 site-specific  integrase [Cronobacter phage  ENT47670] | Phage integrase family [PF00589.24]; Phage integrase, N-terminal SAM-  like domain [PF02899.19]; Phage  integrase, N-terminal SAM-like  domain [PF13495.8] | sp\|P22877\|INTR_SACER Integrase; XhXr |
| 29_S2_k57_103201_flag_0_multi_15.4283_len_102212 |  |  |  |  | Phage integrase family [PF00589.24] |  |
| 29_S2_k57_1882072_flag_1_multi_10_9899_len_47535-cat_2 |  |  | YP_024945.1 | YP_024945.1 putative integrase  protein [Burkholderia virus  BcepC6B] | Phage integrase family [PF00589.24]; Arm DNA-binding domain  [PF13356.8] | sp\|P22877\|INTR_SACER Integrase; XhXr |
| 29_S2_k57_3207518_flag_1_multi_9_0000_len_30771-cat_2 | K14059 | integrase | YP_001552358.1 | YP_001552358.1 integrase  [Mycobacterium phage Giles] | Phage integrase family [PF00589.24] | sp\|P22877\|INTR_SACER Integrase; XhXr |
| 29_S2_k57_3590775_flag_1_multi_21_6770_len_13378-circular-cat_2 |  |  |  |  | Phage integrase family [PF00589.24]; Arm DNA-binding domain  [PF13356.8] | sp\|P22877\|INTR_SACER Integrase; XhXr |
| 29_S2_k57_4504829_flag_0_multi_65_8771_len_43392-cat_2 |  |  |  |  |  | sp\|P22877\|INTR_SACER Integrase; XhXr |
| 29_S2_k57_8184121_flag_0_multi_10.6982_len_13141 |  |  |  |  | Phage integrase family [PF00589.24] |  |
| 29_S3_k57_2770978_flag_0_multi_19.0874_len_62944 |  |  | YP_009194162.1 | YP_009194162.1 putative integrase |  | REFSEQ hypothetical protein; Xu |
| vOTU | Kegg_id | Kegg_hit | Viral_id | Viral_hit | Pfam_hits | Vogdb_description |
| 29_S3_k57_4566374_flag_0_multi_12.9983_len_69651 |  |  |  |  | Phage integrase family [PF00589.24] | sp\|P22877\|INTR_SACER Integrase; XhXr |
| 32_S1_k57_8005552_flag_0_multi_23.1115_len_36384 |  |  | YP_002321479.1 | YP_002321479.1 integrase arm-  type DNA-binding domain-  containing protein  [Stenotrophomonas phage S1] | Arm DNA-binding domain  [PF13356.8]; Phage integrase family [PF00589.24] | sp\|P22877\|INTR_SACER Integrase; XhXr |
| 32_S2_k57_11087896_flag_0_multi_13.9981_len_68123 |  |  | YP_009188643.1 | YP_009188643.1 putative integra-se [Gordonia phage GMA3] | Phage integrase family [PF00589.24] | sp\|P22877\|INTR_SACER Integrase; XhXr |
| 32_S2_k57_11443506_flag_3_multi_31.0006_len_50684 |  |  |  |  | Phage integrase family [PF00589.24] | sp\|P22877\|INTR_SACER Integrase; XhXr |
| 32_S2_k57_771536_flag_0_multi_12.4558_len_24435 | K04763 | integrase/recombinase XerD | YP_009199114.1 | YP_009199114.1 putative recom-binase XerD [Brevibacillus phage Jenst] | Phage integrase family [PF00589.24] | sp\|P22877\|INTR_SACER Integrase; XhXr |
| 32_S3_k57_3088676_flag_1_multi_11.0000_len_46314 |  |  | YP_009012282.1 | YP_009012282.1 integrase  [Mycobacterium phage BigNuz] | Phage integrase family [PF00589.24] | sp\|P22877\|INTR_SACER Integrase; XhXr |
| 32_S3_k57_5168632_flag_1_multi_7.8168_len_37784 |  |  | YP_007112444.1 | YP_007112444.1 integrase [Enterobacterial phage mEp390] | Phage integrase family [PF00589.24] | sp\|P22877\|INTR_SACER Integrase; XhXr |
| 35_S1_k57_2071602_flag_0_multi_25_3611_len_11665-cat_2 | K04763 | integrase/recombinase XerD | YP_009199114.1 | YP_009199114.1 putative recom-binase XerD [Brevibacillus phage Jenst] | Phage integrase family [PF00589.24];  Phage integrase, N-terminal SAM-  like domain [PF02899.19]; Phage  integrase, N-terminal SAM-like  domain [PF13495.8] | sp\|P22877\|INTR_SACER Integrase; XhXr |
| vOTU | Kegg_id | Kegg_hit | Viral_id | Viral_hit | Pfam_hits | Vogdb_description |
| 35_S1_k57_3346215_flag_0_multi_9.6000_len_15680 |  |  |  |  | Phage integrase family [PF00589.24] | sp\|P22877\|INTR_SACER Integrase; XhXr |
| 35_S1_k57_5120045_flag_0_multi_76.0154_len_15370 |  |  | YP_008059154.1 | YP_008059154.1 integrase [Halo-virus HCTV-5] | Phage integrase family [PF00589.24] | sp\|P96629\|INT_BACSU ICEBs1 integrase; Xr |
| 35_S1_k57_5412590_flag_0_multi_74.4810_len_15082 |  |  |  |  | Phage integrase family [PF00589.24] | sp\|P22877\|INTR_SACER Integrase; XhXr |
| 35_S1_k57_6397365_flag_0_multi_72.7471_len_16129 |  |  |  |  | Phage integrase family [PF00589.24];  Phage integrase, N-terminal SAM-  like domain [PF02899.19]; Phage  integrase SAM-like domain  [PF13102.8] | sp\|P22877\|INTR_SACER Integrase; XhXr |
| 35_S1_k57_6397365_flag_0_multi_72.7471_len_16129 |  |  |  |  | Phage integrase family [PF00589.24] |  |
| 35_S2_k57_3598456_flag_0_multi_7.4215_len_10204 |  |  |  |  | Phage integrase family [PF00589.24]; Phage integrase, N-terminal SAM-  like domain [PF13495.8] | sp\|P22877\|INTR_SACER Integrase; XhXr |
| 35_S2_k57_3713203_flag_0_multi_12.5028_len_25743 |  |  |  |  | Phage integrase family [PF00589.24]; Phage integrase, N-terminal SAM-  like domain [PF13495.8] | sp\|P22877\|INTR_SACER Integrase; XhXr |
| 35_S2_k57_6861616_flag_0_multi_13.4062_len_12254 |  |  |  |  | Phage integrase family [PF00589.24] |  |
| 7_S2_k57_11632576_flag_3_multi_13.9753_len_42552 |  |  | YP_009785037.1 | YP_009785037.1 site-specific  integrase | Phage integrase family [PF00589.24] | sp\|P22877\|INTR_SACER Integrase; XhXr |
| vOTU | Kegg_id | Kegg_hit | Viral_id | Viral_hit | Pfam_hits | Vogdb_description |
| 7_S1_k57_7699859_flag_1_multi_7.0000_len_39892 | K04763 | integrase/recombinase XerD | YP_009194042.2 | YP_009194042.2 tyrosine-type  recombinase/integrase [Bacillus  phage vB_BtS_BMBtp3] | Phage integrase family [PF00589.24]; Phage integrase, N-terminal SAM-  like domain [PF02899.19]; Phage  integrase, N-terminal SAM-like  domain [PF13495.8] | sp\|P22877\|INTR_SACER Integrase; XhXr |
| 7_S2_k57_1756606_flag_1_multi_6.5682_len_32454 | K06400 | site-specific DNA recombinase | YP_009953031.1 | YP_009953031.1 serine integrase Mycobacterium phage Curiosium | Resolvase, N terminal domain  [PF00239.23]; Recombinase  [PF07508.15] | sp\|P03014\|PINE_ECOLI Serine recombinase  PinE; Xr |
| 7_S2_k57_350917_flag_3_multi_18.0000_len_39184 | K06400 | site-specific DNA recombinase | YP_009616548.1 | YP_009616548.1 serine integrase [Streptomyces phage Hydra] | Resolvase, N terminal domain  [PF00239.23]; Recombinase  [PF07508.15] | sp\|P03014\|PINE_ECOLI Serine recombinase  PinE; Xr |
| 7_S3_k57_10080475_flag_1_multi_6.4180_len_13590 |  |  | YP_008129787.1 | YP_008129787.1 integrase  [Mycobacterium phage Leo] | Phage integrase family [PF00589.24] |  |
| 7_S3_k57_12141746_flag_3_multi_26.0004_len_61025 |  |  |  |  | Phage integrase family [PF00589.24] | sp\|P22877\|INTR_SACER Integrase; XhXr |
| 7_S3_k57_12141775_flag_3_multi_23.0004_len_59157 |  |  | YP_009784265.1 | YP_009784265.1 putative  integrase/recombinase protein  [Burkholderia phage Bp-AMP1] |  | sp\|P22877\|INTR_SACER Integrase; XhXr |
| 7_S3_k57_7386454_flag_0_multi_59.8804_len_56826 | K14059 | integrase | YP_009952554.1 | YP_009952554.1 tyrosine  integrase [Mycobacterium phage  Amelie] | Phage integrase family [PF00589.24]; Integrase [PF12835.9] | sp\|P22877\|INTR_SACER Integrase; XhXr |
